# Mitochondrial calcium modulates odor-mediated behavioral plasticity in *Caenorhabditis elegans*

**DOI:** 10.1016/j.mocell.2026.100367

**Published:** 2026-05-04

**Authors:** Hee Kyung Lee, Abe Gayle Santos, Kyu-Sang Park, Kyoung-hye Yoon

**Affiliations:** 1 Department of Physiology, Yonsei University Wonju College of Medicine, 20 Ilsan-ro, Wonju, South Korea; 2 Organelle Medicine Research Center, Yonsei University Wonju College of Medicine, 20 Ilsan-ro, Wonju, South Korea; 3 Department of Global Medical Science, Yonsei University Wonju College of Medicine, 20 Ilsan-ro, Wonju, South Korea

**Keywords:** Behavioral plasticity, *Caenorhabditis elegans*, Learning, Mitochondrial calcium uniporter, Neuropeptide

## Abstract

Despite growing understanding of the various roles mitochondria play in neurons, how they contribute to higher brain functions such as learning and memory remains underexplored. Here, using the nematode *Caenorhabditis elegans,* we found that the mitochondrial calcium uniporter (MCU) pore-forming unit MCU-1 is required for aversive learning of specific odors sensed by the AWC sensory neuron. MCU-1 expression was required in the sensory neuron at the time of odor conditioning for proper behavioral response to 60 min of prolonged odor exposure. We discovered that calcium entry into the mitochondria in AWC responds to the length of odor stimulus: calcium is elevated after 60 minutes of odor but not after 30 minutes, suggesting a gating mechanism that can discriminate the duration of sensory stimuli. Through genetic and pharmacological manipulation, we show that calcium influx through the MCU causes mtROS production, leading to NLP-1 secretion from the AWC neuron and odor learning. Overall, our results demonstrate that, by regulating mitochondrial calcium entry, mitochondria can respond to the length of a sensory stimulus to modulate the synaptic response, resulting in context-appropriate learning and behavior.

## Graphical Abstract

**Figure:**
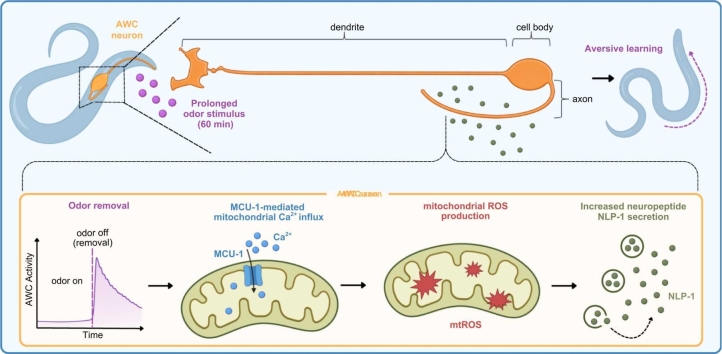

## INTRODUCTION

Learning and memory allow the past experience of an organism to adjust and shape their behavior, thereby increasing its fitness and chance of survival. This behavioral plasticity is modulated by multiple factors such as the length and frequency of the experience, or concurrent external and internal sensory inputs. Such set of factors ensures that the learning and strength of the memory is commensurate to the type of experience and its context.

The cellular and molecular substrate for learning and memory is the change in synaptic activity strength. Among the numerous factors involved in modulating synaptic activity, mitochondria are reported to play a role by supplying ATP through energy metabolism (Ashrafi et al, 2020; Hebert-Chatelain et al, 2016; Rangaraju et al, 2014; Verstreken et al, 2005), buffering calcium (Alnaes & Rahamimoff, 1975; David et al, 1998; Lee et al, 2007; Tang & Zucker, 1997), and producing localized ROS (Jia & Sieburth, 2021; Zhao et al, 2018). Consistent with this, a few studies have shown instances where mitochondria are involved in learning and memory (Duan et al, 2021; Hebert-Chatelain et al., 2016; Oettinghaus et al, 2016), and mitochondrial dysfunction in the brain has been linked to synaptic dysfunction and decline in cognitive abilities (Todorova & Blokland, 2017). Further studies of mitochondria’s role in neuronal function are needed to understand the various ways in which metabolic activities and their dysfunction are interconnected with higher brain functions such as learning and memory.

The mitochondrial calcium uniporter (MCU) complex is the main route of calcium entry into mitochondria. The complex consists of MCU channel protein and several regulatory subunit proteins that play modulatory roles. In *Caenorhabditis elegans*, the complex consists of the channel protein MCU-1, along with 2 regulatory subunit proteins MICU-1 and EMRE-1. With the molecular identity revealed a little more than a decade ago (Baughman et al, 2011; De Stefani et al, 2011), studies are beginning to uncover its various roles in different tissues during normal and pathological conditions (Burkewitz et al., 2020, Higashitani et al., 2023). Even so, the mechanisms by which the channel is modulated in various tissues under physiological conditions remain poorly understood, and its functions and regulation in neurons are still emerging areas of study.

The nematode *C. elegans* displays complex behavior that can be modified through past experience. Its simple nervous system consisting of 302 neurons, its genetic tractability, and the array of tools available offer the chance to investigate the detailed molecular underpinnings of learning and memory. *C. elegans* can learn to increase or decrease its attraction to odors based on past experience and can control the degree of these modifications depending on the context and length of the experience (Colbert & Bargmann, 1995; Kauffman et al, 2010; Lee et al, 2010). For attractive odors such as butanone and benzaldehyde that are mainly detected by the chemosensory neuron AWC, prior experience to the odor in the absence of food makes them less attractive to the worms. How much less is dependent on the length of the experience, and a gradual decline in attraction can be seen from 0 to 2 hours of prior exposure (Lee et al., 2010). Interestingly, this progressive decline is mediated by distinct molecular steps and can be genetically separated. For example, *cng-3* in AWC is required for the mild decrease in attraction after 30 minutes of odor conditioning (O'Halloran et al, 2017). However, aversive learning to 60 minutes conditioning is unaffected in *cng-3* mutants, demonstrating the distinct molecular steps involved to produce the seemingly smooth continual decline in attraction. Similarly, nuclear translocation of the cGMP-dependent protein kinase EGL-4 in AWC is required for aversive learning to 60 minutes or longer conditioning (Lee et al., 2010). Thus, the aversive odor learning paradigm in *C. elegans* offers a compelling system to study the molecular mechanisms of behavioral plasticity.

In this study, we use *C. elegans* to show that MCU facilitates odor learning and thus change in odor behavior by controlling neuropeptide release from the primary sensory neuron. We found that *mcu-1* mutants are defective for aversive learning to AWC^ON^-sensed odors. MCU-1 was required in the AWC neuron at the time of learning. The learning defect was specific to learning after 60-min conditioning, which requires the neuropeptide-like protein NLP-1 neuropeptide. Consistently, calcium influx into the mitochondria only occurred after 60-min conditioning but not after a shorter conditioning time of 30 minutes. By genetic and pharmacological manipulation, we show that MCU in the AWC responds to 60 minutes odor exposure to produce mitochondrial ROS (mtROS), which triggers NLP-1 secretion from the same neuron. Moreover, ectopic activation of MCU or mtROS production during odor exposure was sufficient to accelerate learning. Taken together, our study shows that mitochondrial calcium in neurons contributes to learning by modulating the synaptic response to sensory stimuli.

## MATERIALS AND METHODS

### Worm Cultivation

*Caenorhabditis elegans* were maintained at 20°C on plates containing nematode growth media (NGM) with OP50 bacteria. Unless otherwise specified, day 1 adults were used for experiments. All strains used in this study are listed in Table S1.

### Construction of Plasmids and Transgenic Strains

Whole worm RNA was isolated using the Direct-zol RNA Microprep kit (Zymo Research). cDNA was prepared using SuperScript IV First Strand Synthesis System (Invitrogen). *mcu-1* sequence was amplified from worm cDNA using PrimeSTAR MAX DNA Polymerase (Takara) and cloned into the pCR-Blunt II-TOPO vector (Invitrogen). This was used to amplify *mcu-1* with the appropriate restriction enzyme sites required for the different plasmids. roGFP sequence was amplified from genomic DNA of the KWN119 strain (gift from the Nehrke lab) which expresses mito-roGFP under the *myo-3* muscle-specific promoter. Primers that bind to the 3’ end of the *myo-3* promoter and reverse primer binding to the *unc-54* 3’UTR sequence were used to amplify the whole fragment; then existing enzyme restriction enzyme sites were used to cut the insert, which was ligated into the AWC-specific *ceh-36p* plasmid (gift from Juang lab). MiniSOG sequence was amplified from the genomic DNA of CZ20310 strain (CGC), which expresses HIS-72 tagged miniSOG in the germline. The first 228 nt sequence of *tomm-20,* which includes 165 nt coding sequence and the first intron, was amplified from worm genomic DNA. Venus sequence was cloned from a worm plasmid containing the sequence (gift from the Sieburth lab). *nlp-1* and *nlp-3* sequence were amplified from worm cDNA. CamPARI2(L398T) sequence was cloned from pAAV_hsyn_NES-his-CaMPARI2-L398T-WPRE-SV40, a gift from Eric Schreiter (Addgene plasmid # 101064; http://n2t.net/addgene:101064; RRID:Addgene_101064). All primers used in this study are listed in Table S2.

To generate transgenic worm strains, plasmids were injected at 20 ng/μl, along with 40 ng/μl *unc-122p::gfp* or *mCherry* co-injection marker plasmids. pUC19 plasmid was used to bring the total DNA concentration of the injection mix to 100 ng/μl.

### Chemotaxis Assay

Assay was performed as described previously (Colbert & Bargmann, 1995). For assay plates, 10 mL of agar media (1.6% Difco Granulated Agar (Beckton Dickinson, Franklin Lakes, NJ, USA), 1 mM CaCl_2_, 1 mM MgSO_4_, 5 mM KPO_4_) was poured into 10 cm petri dish and were left out to dry for at least 1 day. Well-fed, day 1 adult worms were used for all assays. Odors were diluted as follows unless otherwise stated: 1 μL Benzaldehyde (Sigma) in 200 μL ethanol, 1 μL 2-Butanone (Sigma) in 1 mL ethanol, 1 μL Diacetyl (Waco) in 5 mL, and 1 μL 2,3-Pentanedione (Alfa aesar) in 10 mL ethanol. For extrachromosomal transgenic strains, we scored only the transgenic animals that expressed the fluorescent co-injection marker.

### Aversive Associative Learning Assay

Day 1 adults were collected from plates and washed 3 times with S-basal buffer. For conditioning, worms were placed in 1 mL of S-basal buffer with or without each odorant with the following concentrations: Benzaldehyde 1:10,000, 2-Butanone 1:10,000, Diacetyl 1:5,000, 2,3-Pentanedione 1:100,000. The microcentrifuge tubes were rotated on a rotator for conditioning and then washed twice with s-basal buffer and once with deionized water before conducting the chemotaxis test. For heat-shock-inducible *mcu-1* mutant worms, synchronized worms at the L1 stage were transferred to seeded NGM plates to grow until ready for heat shock. At each developmental stage, plates of worms were sealed with parafilm and immersed into a 32°C water bath for 1 hr. Plates were moved back to 20°C until day 1 stage when the aversive learning assay was conducted. Worms heat-shocked at the adult stage were placed at 20°C for 2 hours until used for the assay. For mito-miniSOG strains, worms were grown in plates covered with aluminum foil to prevent excess light exposure during development. We found that for learning and NLP-1 secretion, these strains were sensitive to ambient light, and this was sufficient to induce accelerated odor learning and NLP-1::Venus secretion (below). Thus, odor conditioning and subsequent chemotaxis assays were conducted either on the bench (light+) or in a dark room to avoid ROS generation by ambient light (light-).

### Pharmacological Treatment

All pharmacological substances used in this study, except for H_2_O_2_, necessitated permeabilizing the cuticle. This was accomplished by growing synchronized worms in *dpy-8* RNAi bacteria at the last molting stage, from L4 to adult stage. *dpy-8* RNAi bacteria clone was obtained from the Ahringer RNAi library (Kamath et al, 2003) (Source BioScience). RNAi plates were prepared as previously described (Ahringer). Permeabilization of the cuticle did not affect their behavior (data not shown). Following are the stock solutions used: 6.358 mM Ruthenium Red in water (Millipore), 0.5 mg/mL RU360 (Millipore) in deoxygenated water, 50 mM of Kaempferol (Santa Cruz Biotechnology) in EtOH, 50 mM of Juglone (Sigma-Aldrich) in DMSO, 100 mM of N-Acetyl-L-Cysteine (BioBasic) in water, 18.938 mM of mitoTEMPO (Santa Cruz Biotechnology) in DMSO, and 30% H_2_O_2_ (Sigma-Aldrich). For the behavior assay, about 1000 adults worms were transferred into a 1.5 mL Eppendorf tube with S-basal and washed 3 times. Pharmacological treatments were added to washed worms at final concentrations of 100 μM for Ruthenium Red, 0.5 μM for RU360, 100 μM for Kaempferol, 300 μM for Juglone, 0.5 mM for N-Acetyl-L-Cysteine, 10 μM for mitoTEMPO, and 5 H_2_O_2_.

### Microscopy and Analysis

All images were obtained using Zeiss LSM800 confocal microscope with Zeiss C-Apochromat 40x/1.2 W or 60x/1.2 W Korr objective and ZEN 2.6 software. For coelomocyte imaging, 30-40 L4 stage animals were collected from NGM plates, washed 3 times, and exposed to either butanone (1:10,000 in S-basal) for 1 hr or to each pharmacological treatment for 10 minutes in S-basal. After treatment, worms were briefly washed, then paralyzed using sodium azide and mounted on 2% agarose pads. All coelomocytes along the length of the worm was imaged. Maximum intensity projection was obtained using image stacks and used for analysis. Fluorescence was quantified by manually delineating each coelomocyte using the polygon selection tool and measuring the integrated density in ImageJ.

For the mito-miniSOG strain, we found that even ambient light was enough to significantly increase NLP-1::Venus in the coelomocytes. Worms were grown in plates wrapped in foil to prevent excess light exposure, but light exposure was inevitable during microscopic analyses. We also confirmed that, although ambient light produces ROS, only strong blue light (30 minutes) was able to induce AWC ablation (Fig. S3).

To image mito-roGFP and calculate reduction/oxidation ratios, worms were immobilized in 30 mg/mL 2,3-butanedione 2-monoxime (BDM). Worms were imaged at 2 different wavelengths for excitation, 488/405 for the reduced/oxidized (R/O) ratio (Vevea et al, 2013). Maximum intensity projection was obtained for each image. ROI was set in the green channel by using the polygon tool to select each subcompartment, then thresholding and using the “Analyze Particles” option in ImageJ. Fluorescence intensity of the reduced and oxidized mitochondria to obtain the ratio of 2 stacks after subtracting the background value from the 2 values. Average value was obtained for the subcompartment by multiplying the mean and area value for each particle within the subcompartment, and the sum was divided by total Area. To check the upper range of roGFP redox ratios, treatment with 5 H_2_O_2_ for 10 min was used as positive control.

### Calcium Imaging and Analysis

For GCaMP imaging of cytoplasmic calcium, worms were immobilized in a poly(dimethylsioloxane) (PDMS) microfluidic chamber described previously (Chronis et al, 2007). Using the microfluidic chamber design generously provided by the Bargmann lab, the SU-8 micro mold and PDMS chip were constructed by MicroFit (Hanam, Republic of Korea). Day 1 adult worms were transferred to unseeded plates to clean bacteria off the body, then transported to the chamber in S-basal buffer via polyethylene tubing (Intramedic). Benzaldehyde at 1:100,000 dilution was used for imaging. GCaMP fluorescence was visualized at 40x magnification using IX-73 inverted fluorescent microscope (Olympus) attached to Iris 9 sCMOS camera (Photometrics) and a pE-800 LED illuminator (CoolLED). Fluorescence was recorded using MetaFluor 3.1 at approximately 5 frames/s. Resulting image sequence was processed using the Fiji software (NIH). ROI was aligned using the ‘align slices in stack’ function of the Template Matching plugin, and then pixel intensity of AWC cell body was measured.

For CaMPARI2 imaging, worms in S-basal buffer or odor solution that had sunk to the bottom of the tube was aspirated and transferred to an empty NGM plate. Extra buffer was added to dilute the odor (odor removal), and the plate was immediately irradiated with 405 nm blue light for 5 minutes. Worms were immobilized with sodium azide and mounted on 2% agarose pads for confocal imaging. Maximum intensity projection was obtained using image stacks and used for analysis. ROI was set and red/green ratio was calculated in the same way as roGFP.

### Statistical Analysis

All data are expressed as means ± standard error of the mean (SEM). Statistical significance was determined by student's *t* test or one-way ANOVA. Asterisks indicate: *P < .05, **P < .01, ***P < .001, and ****P < .0001. All experiments were performed in at least 3 independent trials on different dates.

## RESULTS

### *mcu-1* Mutants Display AWC^ON^-Specific Aversive Learning Defect

**Fig. 1:**
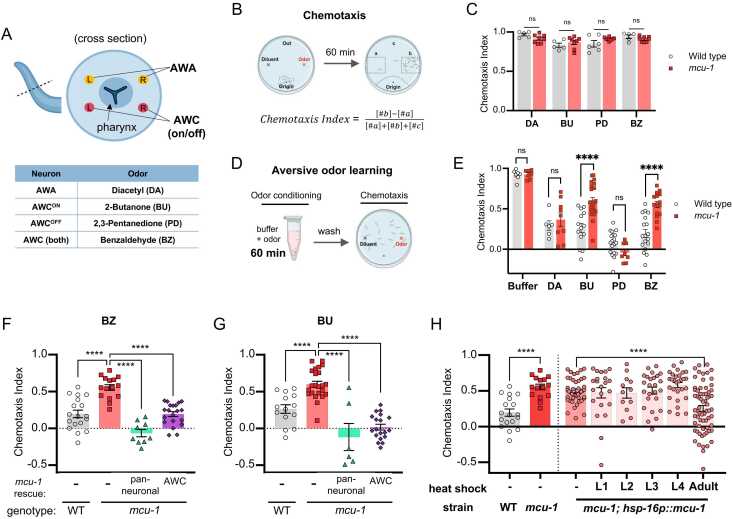
*mcu-1 is required in the AWC^ON^ sensory neuron at the adult stage for aversive odor learning.* (A) Each *C. elegans* chemosensory neuron pairs detect distinct attractive odorants. (B) Schematic for chemotaxis assay and chemotaxis index (CI) calculation. (C) Chemotaxis index for naïve N2 and *mcu-1(tm6026)* worms. (D) Schematic for aversive odor learning. (E) Chemotaxis index in worms after 60 min odor conditioning. (F,G) Aversive odor learning in neuron(*rab-3p*)- and AWC(*ceh36p*)-specific rescue strains for benzaldehyde(BZ) and butanone(BU). (H) Chemotaxis index of transgenic strains expressing MCU-1 under the heat-shock-inducible promoter (*hsp-16.2p*). Worms were given heat shock at different larval stages and tested as day 1 adults. P values were determined using Student’s *t* test (C, E, comparison between WT and *mcu-*1 in H) or one-way ANOVA with Dunnett’s multiple comparisons test (F, G, comparison between larval-stage-heatshock in H). Asterisks indicate *P* value (ns, not significant; ****P < .0001).

To study the role of MCU in neurons, we tested a null mutant strain of *mcu-1* for behavioral defects, including odor behaviors. *Caenorhabditis elegans* detects attractive volatile odors mainly through 2 pairs of sensory neurons, AWC and AWA. The AWC neuron pair is asymmetric and expresses different sets of receptors (Wes & Bargmann, 2001). With this in mind, we selected odorants that represented the function of each sensory neurons: diacetyl, 2-butanone, 2,3-pentanedione, and benzaldehyde, which are mainly sensed by AWA, AWC^ON^, AWC^OFF^, and both AWCs, respectively (Fig. 1A). Mutant worms showed normal chemotaxis for all odorants tested, indicating that worms can sense and move toward attractive odors in the absence of MCU-1 (Fig. 1B-C).

We next tested *mcu-1* mutants for odor-learning behaviors. In the aversive odor learning paradigm, previous exposure to odors without food causes worms to ignore or avoid what used to be an attractive odor (Fig. 1D). After 60 minutes of pre-exposure, *mcu-1* mutants failed to display aversive learning to benzaldehyde and butanone, whereas learning for other odors remained normal (Fig. 1E). Since butanone is sensed by AWC^ON^ and benzaldehyde is sensed by both AWC neurons, this suggested that learning was specifically defective for AWC^ON^. Consistent with this, pentanedione, which is sensed by the AWC^OFF^ neuron, showed normal aversive learning. Thus, *mcu-1* mutants fail to learn odors sensed by the AWC^ON^ neuron.

### MCU-1 Is Required in the Primary Sensory Neuron for Aversive Odor Learning

We next sought to find where MCU-1 was needed for odor learning. We hypothesized that MCU-1 is likely needed in the neurons. To test this, we generated a transgenic strain that expressed MCU-1 under a neuron-specific promoter (*rab-3p*). As expected, neuronal *mcu-1* rescue strain showed normal aversive learning to both butanone and benzaldehyde (Fig. 1F,G). To further narrow down the neuron responsible, we next made a cell-specific *mcu-1* transgenic rescue strain. We hypothesized that MCU-1 is likely needed in the neuronal circuit that includes the primary sensory neuron AWC and a few immediately downstream interneurons (Chen et al., 2006, White et al., 1986). When we expressed MCU-1 under the AWC-specific promoter (*ceh-36p*), we found that it was also sufficient to fully restore aversive learning (Fig. 1F,G). Thus, MCU-1 is needed in the sensory neuron AWC to mediate odor learning.

### MCU-1 Is Required Post-Developmentally

We next wondered when MCU-1 is needed for odor learning. *Caenorhabditis elegans* goes through 4 larval stages until they reach adulthood. For most neurons, cell fates are determined during the embryonic stage, and neurons are fully functional by the time of hatching. However, the strength of neuronal response may change as the worm grows and matures (Fujiwara et al, 2016). To determine at which stage MCU-1 is needed for odor learning, we generated a transgenic strain that expressed MCU-1 under a heat-inducible promoter. Inducing MCU-1 expression at different larval stages revealed that aversive learning was restored only when MCU-1 was induced at the adult stage, which was only a few hours before they were used for the aversive learning assay (Fig. 1H). This showed that the learning defect is not due to a developmental defect and that MCU-1 expressed in the adult stage is sufficient to rescue learning. In addition, imaging fluorescently labeled AWC neurons revealed no change in morphology from wild type to mutant (Fig. S1), adding to the evidence that the role of MCU-1 in odor learning is post-developmental. Notably, inducing MCU-1 during the larval stages did not rescue learning in adults, which may indicate high turnover rate of the MCU-1 protein. The lack of rescue may be explained if the proteins induced earlier are no longer present by the adult stage. Taken together, MCU-1 is required at the adult stage for odor learning.

### Aversive Learning Defect in *mcu-1* Is Specific to 60 Minutes Conditioning

In aversive odor learning, attractiveness to an odor progressively declines with longer pre-exposure, or conditioning, time (Lee et al., 2010). For AWC-mediated odor learning, some of these steps are molecularly distinct: learning to 30 minutes conditioning requires *cng-3* (O'Halloran et al., 2017), and further decrease in the chemotaxis index (CI) after 60 minutes or more requires translocation of the cGMP-dependent protein kinase EGL-4 into the nucleus (Lee et al., 2010).

**Fig. 2:**
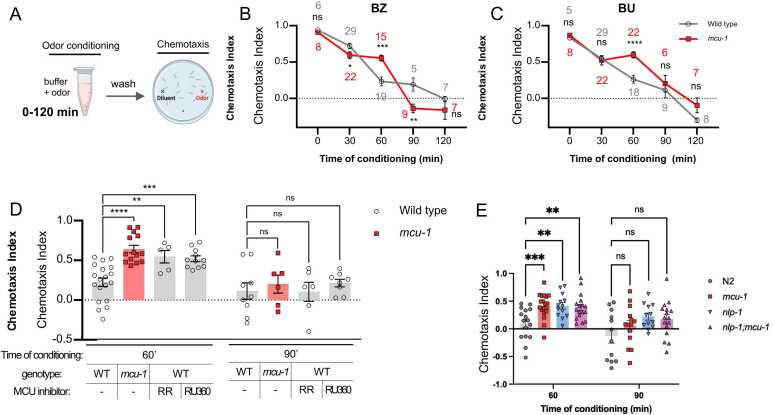
*Mutants of mcu-1 are specifically defective for 60 minutes odor learning.* (A) Aversive odor learning was conducted with varied conditioning times. (B,C) Chemotaxis index after different conditioning times (number above or below the data point indicates number of trials). (D) Wild type worms treated with pharmacological blockers of MCU, ruthenium red(RR) and RU360, display the same learning defect to butanone as *mcu-1* mutants. (E) *nlp-1* mutant is also defective for aversive learning after 60 minutes conditioning but not after 90 minutes. *P* values were determined using Student’s *t*-test (B,C,E) or one-way ANOVA with Dunnett’s multiple comparisons test (D). Asterisks indicate *P* value (ns, not significant; *P < .05; **P < .01; ***P < .001; ****P < .0001).

To further characterize the learning defect of *mcu-1* mutants, we tested different conditioning times ranging from 30 to 120 minutes (Fig. 2A). At 30 minutes conditioning, both wild type and *mcu-1* mutant strain showed a modest decrease in attraction (Fig. 2B,C). At 60 minutes pre-exposure, wild type showed further decrease in CI, whereas *mcu-1* mutants remained at the 30 minutes level. However, after 90 minutes and beyond, both wild type and *mcu-1* worms similarly displayed a much lower CI close to 0. The pattern was observed for both butanone and benzaldehyde (Fig. 2B,C). Previously, it was shown that the protein kinase G EGL-4 in AWC moves the nucleus in response to prolonged odor exposure and that this was required for aversive odor learning (Lee et al., 2010). To see whether the absence of MCU-1 diminished learning by preventing EGL-4 nucleus translocation, we assessed the location of EGL-4 using a transgenic strain that expressed GFP-tagged EGL-4 in the AWC (Lee et al., 2010). We found that EGL-4 nuclear translocation occurred normally in response to odor exposure in both wild-type and mutant background (Fig. S2). This indicates that MCU-1 represents an independent step in the gradual decline in attraction during odor learning. Thus, to our surprise, the learning defect was specific to 60 minutes conditioning but dispensable for shorter and longer conditioning times.

### MCU Function Is Required at the Time of Conditioning

Earlier, we found that MCU-1 expression is required post-developmentally for odor learning. To further narrow down when MCU-1 function is needed, we used ruthenium red and RU360, pharmacological blockers of MCU (Ying et al, 1991). Because the *C. elegans* cuticle prevents the uptake of most pharmacological agents, we permeabilized the worm cuticle by knocking down a gene (*dpy-8*) involved in cuticle formation to ensure RU360 uptake (see Materials and Methods) (Sandhu et al, 2021). When RU360 was added to the odor solution during conditioning, we found that both ruthenium red and RU360 mimicked the *mcu-1* mutant phenotype: treatment during 60 minutes pre-exposure inhibited odor learning, but treatment during 90 min pre-exposure yielded normal odor learning indistinguishable from wild type (Fig. 2D). Thus, pharmacological blockers of MCU can be used to reproduce *mcu-1* mutant phenotype, and MCU-1 function is needed at the time of conditioning for successful odor learning.

### MCU Mediates NLP-1 Neuropeptide Secretion From AWC

While it was surprising that MCU-1 was required for learning to a specific length of conditioning time, a similar learning defect had been described previously for another mutant. Chalasani et al had reported that mutant of a neuropeptide gene *nlp-1* showed defective aversive learning for isoamyl alcohol, another AWC-sensed odor (Chalasani et al, 2010). Interestingly, the learning defect was also specific for 60 minutes conditioning, as mutants showed normal learning for 90 minutes conditioning (Chalasani et al., 2010). NLP-1 is expressed in many neurons, including AWC (Nathoo et al, 2001; Taylor et al, 2021). Conducting the same aversive learning experiment on *nlp-1* mutants, but with butanone, confirmed their results (Fig. 2E). We generated double mutants of the 2 genes and found they were also defective for 60 minutes learning, with similar CI as the single mutants. This suggested that the 2 genes are likely acting in the same pathway.

**Fig. 3:**
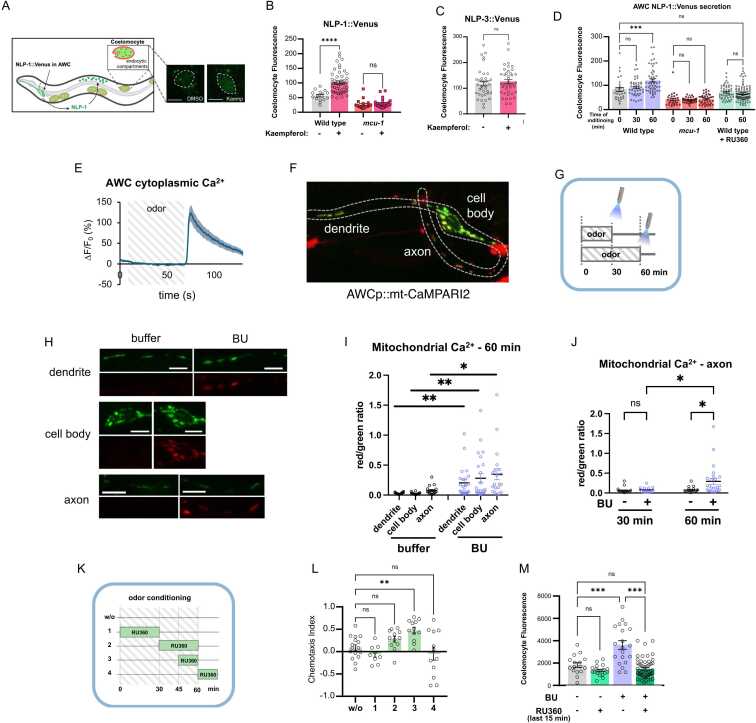
*MCU activation or butanone exposure causes NLP-1 secretion from AWC neurons.* (A) Diagram of NLP-1 secretion from AWC and uptake by coelomocytes. (B) NLP-1::Venus fluorescence measured in coelomocytes with and without 10 minutes treatment with the MCU activator kaempferol. (C) NLP-3:Venus fluorescence in coelomocytes with and without 10 minutes kaempferol treatment. (D) NLP-1::Venus fluorescence in coelomocytes after butanone conditioning. (E) Cytoplasmic calcium response to odor addition and removal in the AWC neuron using GCaMP (*n* = 12). (F) CaMPARI2 expression in AWC mitochondria. (G) Worms were treated with 5 minutes of blue light after 30 minutes or 60 minutes odor exposure. (H) Representative picture of green to red photoconversion after buffer or odor exposure in each subcellular compartment of the AWC neuron. (I) Quantification of photoconversion in each cellular subcompartment. One dot represents the average red to green ratio of all mitochondria found within the subcompartment of one animal. (J) CaMPARI photoconversion in AWC axonal mitochondria in response to different duration of butanone exposure. (K) Diagram describing the different RU360 treatment durations. (L) Effect of RU360 treatment at different time points during the 60 minutes butanone conditioning on odor learning. (M) NLP-1 secretion in worms treated with RU360 during the last 15 minutes of 60 minutes butanone conditioning. For all graphs, each dot represents individual trials. *P* values were determined using Student’s *t*-test (B,C,I,J) or one-way ANOVA with Dunnett’s multiple comparisons test (D,L,M). Asterisks indicate *P* value (ns, not significant; *P < .05; **P < .01; ***P < .001; ****P < .0001).

How may *mcu-1* and *nlp-1* act in the same pathway to mediate odor learning? One likely possibility was that MCU-1 in the AWC is involved in NLP-1 secretion from the same neuron. To test this, we generated a transgenic strain that expressed Venus-tagged NLP-1 in the AWC neurons. Although not functional, the fluorescently tagged NLP-1 is secreted into the body cavity and accumulates in the scavenging coelomocytes, whose fluorescence can be used as a proxy for neuropeptide secretion (Fig. 3A) (Sieburth et al, 2007).

We first tested whether activating MCU causes NLP-1 secretion. For this, we treated wild-type worms with a MCU activator, kaempferol (Montero et al, 2004). After 10 minutes treatment with kaempferol, we found a significant increase in the fluorescent signal in the coelomocytes (Fig. 3B). Kaempferol treatment in *mcu-1* mutants, as expected, did not result in fluorescence increase, showing that activation of MCU, and thus likely calcium influx into the mitochondria, is required for NLP-1 release (Fig. 3B). Interestingly, MCU activation does not result in increased secretion of all neuropeptides from AWC, as no change in fluorescence was observed when another highly expressed neuropeptide, NLP-3 secretion, was monitored (Fig. 3C).

### Prolonged Odor Exposure Leads to NLP-1 Secretion From AWC

If ectopic activation of MCU results in NLP-1 secretion, can we observe the same in response to a natural stimulus, such as 60 minutes odor conditioning? To find out, we exposed the NLP-1::Venus transgenic strain to butanone for either 30 or 60 minutes. We found that wild-type worms exposed to butanone for 60 minutes showed increased fluorescence in the coelomocyte, whereas no increase was observed after 30 minutes exposure (Fig. 3D). This indicates that NLP-1 is secreted only after prolonged odor exposure, and 30 minutes is insufficient. When we tested *mcu-1* mutant worms, they did not show any increase in fluorescence in response to butanone regardless of exposure time (Fig. 3D). These results were all consistent with the learning defect of *nlp-1* and *mcu-1* mutants, which are defective only for aversive learning to 60 minutes conditioning.

Does NLP-1 secretion in response to prolonged odor conditioning require MCU function? To find out, we treated NLP-1::Venus worms with the MCU blocker RU360 during 60 minutes odor conditioning. In the presence of the MCU blocker, there was no increase in fluorescence in the coelomocytes (Fig. 3D). Taken together, our results show that odor exposure of 60 minutes, but not 30 minutes, causes secretion of NLP-1 from AWC in an MCU-dependent manner.

### Prolonged Odor Exposure Leads to Calcium Influx Into the Mitochondrial Matrix

Our results so far suggest that prolonged odor exposure of 60 minutes causes NLP-1 secretion through MCU activation. This implies that calcium influx into the mitochondrial matrix happens only after prolonged odor exposure. To directly observe whether this is the case, we generated a transgenic strain expressing the optogenetic photoconverting calcium indicator CaMPARI2 in the mitochondrial matrix (Figs. 3F, S3). In the presence of elevated Ca^2+^, CaMPARI2 photoconverts from green to red fluorescence when illuminated with 405 nm UV light (Moeyaert et al, 2018). Using this strain, we set out to compare mitochondrial calcium levels after 30 min or 60 min odor exposure. One thing to consider is that the AWC sensory neuron is an “odor-OFF” neuron—it is inhibited during odor stimulus and activated upon odor removal (Fig. 3E) (Chalasani et al, 2007). Therefore, UV irradiation was timed for immediately after the 30- or 60-minutes odor exposure (Fig. 3G).

When we assessed CAMPARI2 photoconversion in the AWC, we found that red fluorescence was found when worms were conditioned for 60 minutes (Fig. 3H-I). Elevation in Ca^2+^ could be observed for all cellular subcompartments, although axonal mitochondria showed the strongest increase (Fig. 3H-I). Strikingly, no change in Ca^2+^ levels were observed after 30 minutes odor exposure (Fig. 3H). This was consistent with the odor-induced NLP-1 accumulation in the coelomocytes (Fig. 3D), strengthening the link between MCU activation, mitochondrial calcium, and NLP-1 secretion.

### NLP-1 Is Secreted Upon Odor Removal

Because AWC is an “OFF” neuron, both cytoplasmic and mitochondrial calcium influx occur upon odor removal. This also establishes the timing of NLP-1 secretion to the time of odor removal. To further confirm that NLP-1 secretion occurs not during but after odor exposure, we used RU360 to block MCU at specific time points during odor conditioning and tested their behavior (Fig. 3K-L). Adding and removing MCU inhibitor RU360 in the first or last half of pre-exposure showed that blocking MCU during the second half of odor conditioning tended to suppress aversive learning (Fig. 3K-I). Furthermore, blocking MCU function during the last 15 minutes was sufficient to prevent odor learning. This suggested that MCU function was required at the end of odor exposure, which coincides with the timing of the cytoplasmic and mitochondrial calcium entry upon odor removal (Fig. 3E, J). When RU360 was added during the washing step immediately after odor exposure, it had no effect on odor learning (Fig. 3K-L). This was not surprising, as there is likely a significant time delay for the inhibitor to reach the neuron.

Next, we tested whether adding RU360 at the end of odor conditioning also prevented NLP-1::Venus secretion. Consistent with their chemotaxis behavior, worms supplemented with RU360 during the last 15 minutes of odor conditioning failed to show NLP-1::Venus accumulation in the coelomocytes (Fig. 3M). Taken together, our data show that prolonged odor exposure causes mitochondrial Ca^2+^ influx upon odor removal, prompting NLP-1 secretion from the AWC, leading to odor learning.

### MCU Activation and Odor Exposure Causes mtROS Production

How does MCU activation and mitochondrial calcium signal NLP-1 release? Previous studies show that calcium influx into the mitochondria stimulates ATP production as well as ROS production, both of which can support synaptic signaling (Jia & Sieburth, 2021; Rangaraju et al., 2014). Since a previous *C. elegans* study had shown that *mcu-1* was required for mtROS-mediated neuropeptide secretion from a pair of interneurons, we wondered whether NLP-1 secretion from the AWC sensory neuron was occurring through a similar mechanism (Jia & Sieburth, 2021).

**Fig. 4:**
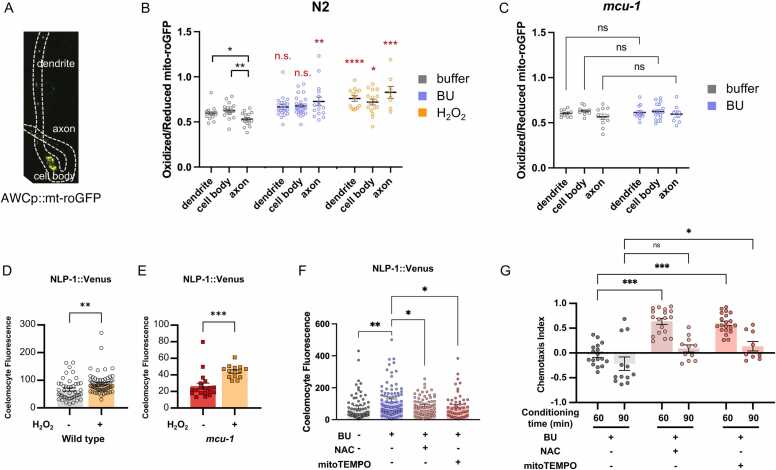
*Odor conditioning causes mtROS production in AWC.* (A) roGFP expression in AWC mitochondria (B) Redox ratio in N2 after exposure to 10 H_2_O_2_ or 60 min butanone. (C) Redox ratio in *mcu-*1 mutants after exposure to 60 minutes butanone. (D,E) 10 min treatment with H_2_O_2_ is sufficient for NLP-1 secretion in both wild type and *mcu-1* mutant. (F) NLP-1 secretion is prevented by treatment with the antioxidants NAC and mitoTEMPO during odor conditioning. (G) Antioxidant treatment inhibits 60 minutes aversive odor learning but not 90 minutes learning. *P* values were determined using Student’s *t*-test (D,E) or one-way ANOVA with Dunnett’s multiple comparisons test (B,C,G). Asterisks indicate *P* value (ns, not significant; *P < .05; **P < .01; ***P < .001).

To find out whether 60 minutes butanone exposure also increased oxidation in the mitochondria, we generated a transgenic strain that expressed the mitochondrially targeted fluorescent ROS sensor mito-roGFP in the AWC neurons (Fig. 4A). We exposed the transgenic strain with butanone for 60 minutes. We also exposed the strain to H_2_O_2_ for 10 minutes as positive control. When treated with H_2_O_2_, oxidation increased in mitochondria of all subcompartments (Fig. 4B). Interestingly, in response to 60 minutes butanone, only mitochondria in the axons showed increased oxidation (Fig. 4B). On the other hand, when mito-roGFP was tested in *mcu-1* mutants, no increase in oxidation was observed in response to the odor (Fig. 4C). Thus, the prolonged odor stimulus produced ROS at the mitochondria of the AWC sensory neuron through MCU-1.

### mtROS Is Required for NLP-1 Secretion and Odor Learning

Superoxide anions are the most abundant ROS produced by the mitochondria and is quickly converted into H_2_O_2_ by superoxide dismutase (Zorov et al, 2014)·H_2_O_2_ can alter activities of target enzymes by modifying the thiols of cysteine residues (Veal et al, 2007). Therefore, we next asked whether adding H_2_O_2_ would cause NLP-1 secretion without the prolonged odor exposure. To do this, NLP-1::Venus worms were treated with 5 mM H_2_O_2_ for 10 minutes and coelomocyte fluorescence was measured. We found that 10-minute H_2_O_2_ treatment was sufficient to induce NLP-1 secretion (Fig. 4D). Moreover, H_2_O_2_ treatment of the *mcu-1* mutant was also sufficient to induce NLP-1 secretion (Fig. 4E). This contrasts with the earlier kaempferol treatment, which was not effective in *mcu-1* mutants (Fig. 3B). This shows that H_2_O_2_ production occurs downstream of MCU activation and calcium influx into the mitochondria.

Next, we reasoned that if mtROS is required for NLP-1 secretion, then treatment with antioxidants that neutralize ROS should prevent its secretion. We therefore treated worms with N-acyl cysteine (NAC), a general antioxidant, or mitoTEMPO, which specifically scavenges mitochondrial ROS, during odor conditioning. Consistent with our hypothesis, worms treated with either NAC or mitoTEMPO failed to show NLP-1::Venus accumulation in the coelomocytes (Fig. 4F).

Finally, we tested whether NAC and mitoTEMPO were also effective in preventing odor learning at 60 minutes. Consistent with NLP-1 secretion, worms treated with NAC and mitoTEMPO showed defective 60 minutes learning but normal 90 minutes learning (Figure 4G) Thus, mtROS is required for NLP-1 secretion, as well as 60-minute odor learning.

### Coincidental Activation of MCU and Odor Exposure Accelerates Odor Learning

**Fig. 5:**
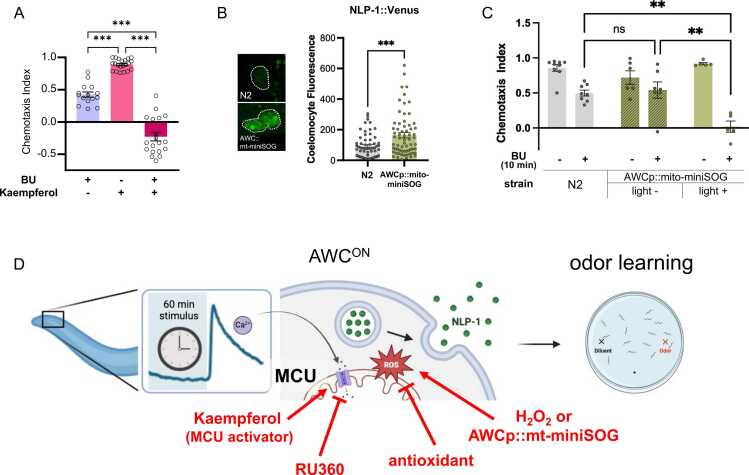
*Coincident mtROS production and odor exposure is sufficient for odor learning.* (A) Kaempferol treatment during 10 minutes odor conditioning results in strong learning. (B) Expression of mitochondrially targeted miniSOG in the AWC neuron is sufficient for NLP-1 release under ambient light. (C) Change in chemotaxis index after 10 minutes odor conditioning. Activation of miniSOG by light causes fast odor learning. (D) Model of MCU activation in response to prolonged odor stimulus, which results in NLP-1 secretion and odor learning. *P* values were determined using one-way ANOVA with Dunnett’s multiple comparisons test. Asterisks indicate *P* value (ns, not significant; **P < .01; ***P < .001).

Our data so far suggest that prolonged exposure to AWC^ON^-specific odors cause MCU activation, causing calcium influx and mtROS production, which is required for 60-minute odor learning. Based on this, we hypothesized that if we ectopically induce mtROS during odor conditioning, we could speed up learning. Normally, 10 minute of butanone conditioning results in only a slight decrease in CI (Fig. 5A). Also, worms treated with kaempferol alone showed CI that are no different from that of naïve worms. This indicates that although kaempferol can cause the release of NLP-1 from AWC, this alone is not enough for learning to occur. On the other hand, when both odor and kaempferol were given, worms exhibited a far lower CI that is typical of 60-minute conditioning (Fig. 5A).

Next, to achieve precise mtROS generation only in the AWC neuron, we expressed the light-activated singlet oxygen generator, miniSOG, in the AWC mitochondria. When illuminated by blue light, ROS produced by miniSOG causes cell death in the cells that express it, making it a useful tool for cell ablation (Fig. S3). However, shorter illumination can be used for ROS generation without causing cell ablation (Jia & Sieburth, 2021). In fact, we found that ambient light was enough for neuropeptide secretion—when transgenic strain was crossed with the nlp-1::Venus strain, we found that the double transgenic strain showed markedly higher Venus fluorescence in the coelomocytes (Fig. 4B). Venus fluorescence could clearly be seen in phagocytic vesicles of the coelomocytes, in contrast to the much subtle fluorescence seen in kaempferol- or H_2_O_2_-treated worms (Fig. 3, Fig. 4). Thus, AWC-specific mtROS can induce NLP-1 secretion from the same neuron.

Lastly, we used the same strain to test for learning behavior. Because ambient light was enough to cause neuropeptide secretion, we tested chemotaxis in a low-light dark room condition versus ambient light condition. Here we also found that activation of mito-miniSOG in AWC neurons produced a sharp decline in CI that is close to zero (Fig. 5C). The same procedure conducted without light showed CI similar to that of the wild type (Fig. 5C). As is the case for kaempferol-treated worms, mito-miniSOG worms conditioned in buffer without odor showed normal attraction, demonstrating that odor must be paired with mtROS for learning to occur (Fig. 5C). Taken together, when odor stimulus is paired with MCU activation and mtROS production, which in turn causes NLP-1 secretion, aversive odor learning is accelerated.

## DISCUSSION

Mitochondrial dysfunction is known to accompany many psychiatric, neurological, and neurodegenerative disorders. However, mechanistic studies on how the dysfunctions contribute to these disorders are lacking. Moreover, there is also a lack of understanding of how mitochondria contribute to normal brain function. Our findings identify a specific aspect of mitochondria function, which, by control of selective calcium influx in response to stimuli and mtROS production, contributes to normal brain function and behavior.

Our study shows that MCU-1, and thus mitochondrial calcium, contributes to behavioral plasticity in *C. elegans* by responding to prolonged sensory stimuli and regulating neuropeptide release at the sensory neuron. We found that *mcu-*1 mutants are defective for 60 minutes aversive odor learning and that this was due to lack of NLP-1 secretion that normally occur after 60 minutes odor conditioning. Calcium influx into the mitochondria and NLP-1 secretion only occurred after 60 minutes of odor stimuli but not after shorter 30 minutes stimuli, which exactly mirrored the 60-minute-specific *mcu-1* learning defect. This hints at a mechanism to selectively activate MCU in response to prolonged odor conditioning to produce the appropriate decrease in attraction and therefore learning. Calcium influx into mitochondria has been shown to produce mtROS (Doser and Knight, 2023, Jia and Sieburth, 2021), and we showed that in the AWC neuron, prolonged odor exposure causes calcium influx and mtROS production, which is abolished in the *mcu-1* mutant. Consistent with the above model, premature activation of MCU or ectopic mtROS during odor exposure accelerated learning. The study shows that mitochondria in neurons play a direct role in determining the composition of synaptic signals during neuronal activity—whether to release a specific neuropeptide in addition to the classical neurotransmitter. The release of this neuropeptide alters the animal’s behavior to display the appropriate level of response to the experienced odor.

A previous study in Drosophila reported odor memory defects in MCU mutant flies (Drago & Davis, 2016). However, in flies, MCU was required for the proper development of the mushroom body neurons, and thus silencing MCU after pupation had no effect on memory. This demonstrates the many roles mitochondria and mitochondrial calcium play in different systems and organisms and highlight the benefit of studying gene function in multiple model organisms.

### MCU Responds to Conditioning Duration

Since its molecular identity was discovered, studies have identified various regulatory mechanisms of MCU function. In addition to the pore-forming subunit MCU-1, the MCU complex include auxiliary subunits MICU and EMRE that regulate channel activity. MICU senses calcium through its EF-hand domain, causing MCU to open in response to cytoplasmic calcium increase (Vais et al, 2020). Consistently, simultaneous imaging of cytoplasmic and mitochondrial calcium in neurons show mitochondrial calcium influx occurring concurrently with cytoplasmic calcium increase (Katsenelson et al, 2022).

In addition to MICU and EMRE, additional factors were found to regulate MCU activity in mice studies, such as CAMKII, MCUR1, or mitochondrially localized IGFR, suggesting that MCU responds to multiple cues in addition to cytoplasmic calcium concentration (Katsenelson et al., 2022; Lin et al, 2019; Vais et al, 2015). This is consistent with the emerging view that MCU-mediated calcium influx is selective, occurring in response to more significant changes in cellular activity. Multiple studies show that mitochondrial calcium influx is usually coupled to either stronger electrical stimulation of the cell or localized cytoplasmic calcium increase (Alvarez-Illera et al, 2020; Csordas et al, 2013; Doser et al., 2023; Groten & MacVicar, 2022; Katsenelson et al., 2022; Lin et al., 2019; Stoler et al, 2022).

Our results indicate that MCU activity in the AWC neuron represents a threshold for the difference between 30 and 60 minutes aversive odor learning. This suggests that MCU, and hence mitochondrial calcium entry, may act as a duration sensor for sensory conditioning (Fig. 5D). How may this occur? Contrary to most other sensory stimuli, extended odor stimulation in the AWC is characterized by prolonged inactivity rather than activity, due to its OFF-neuron characteristics (Fig. 3E). This rules out the progressive buildup of MCU activity and mitochondrial calcium or slow accumulation of secreted NLP-1 during the extended stimulus. Instead, our evidence points to a one-time binary response. The nature of the signal that regulates MCU activity in response to stimulus length remains to be seen. Previously, a careful characterization of AWC calcium response showed that progressively longer odor exposure time from 10 seconds, 1 minute, to 3 minutes resulted in a progressively faster and higher calcium spike at the synaptic terminal upon odor removal (Ventimiglia & Bargmann, 2017). This shows that cytoplasmic calcium at the synapse can reflect the duration of odor stimulation. Whether this also applies to 30 and 60 minutes odor exposure and whether this is associated with the calcium entry we observe in the mitochondria will be an interesting direction for future study.

Despite the gradual decline in attraction in response to longer conditioning times, it is noteworthy that 60 minutes aversive odor learning is not a prerequisite for the further decrease in CI for 90 minutes learning. This shows that several independent molecular steps are responsible for the gradual decline. Our study shows that in addition to *cng-3* and EGL-4 nuclear translocation, mitochondrial calcium influx through the MCU can also be included as one of these steps. Instead of relying on one mechanism to modify behavior in increasing magnitude, aversive odor learning is the result of several independent and often redundant mechanisms. Similarly, aversive salt learning in *C. elegans* also display time-dependent change in avoidance, with different genes involved for 10 min versus longer conditioning times (Dekkers et al, 2021).

### Compartment-Specific Functions of Mitochondria

In a cell with polarized morphology and compartment-specific functions, such as the neuron, mitochondria also have compartment-specific morphology and functions (Seager et al, 2020). At the synapse, mitochondria contribute to synaptic signaling both in the presynaptic and postsynaptic ends. At the presynaptic terminus, mitochondria are needed to generate sufficient ATP to fuel synaptic vesical release as well as vesicle recycling (Ashrafi et al., 2020). They also buffer cytoplasmic calcium at the presynaptic terminus, affecting neurotransmission (Kwon et al, 2016). At the postsynaptic side, mitochondria can modulate the strength of the synaptic input by buffering cytoplasmic calcium (Hirabayashi et al, 2017) or by recruiting AMPA/GLU-1 receptors (Doser et al., 2023).

For neuropeptide secretion, previous studies have shown that mitochondria in the axon, and more specifically, in the synaptic terminal are important for synaptic transmission (Jia and Sieburth, 2021, Zhao et al., 2018). Although our study does not directly address whether it is the axonal mitochondria that is specifically acting in NLP-1 secretion, we were able to assess calcium influx and mtROS increase in each cellular compartment: we found that whereas calcium influx after 60 minutes odor exposure is observed in all cellular compartments (Fig. 3I), significant mtROS increase occurred more prominently in the axons (Fig. 4B). This may suggest compartment-specific mechanism in mitochondria to modulate the amount of mtROS produced in response to calcium influx. Further studies on the roles of mitochondria in specific compartments will reveal the mechanism of how calcium influx does and does not result in mtROS increase to regulate the downstream signaling cascade.

### mtROS and Neuropeptide Release

In *C. elegans* neurons, it was previously shown that mitochondrial H_2_O_2_ increases the release of the neuropeptide FLP-1 from the AIY neuron, which signals to peripheral tissues to induce the oxidative stress response (Jia & Sieburth, 2021). The study showed that different bacterial diets, thus internal metabolic cues, can cause mitochondrial calcium influx, as well as mtROS and H_2_O_2_ production, from the AIY axonal mitochondria. Our study adds to the evidence that this mechanism of neuropeptide secretion may be a general process that occurs in other neurons in *C. elegans* and in response to a wider variety of cues beyond metabolic, such as odor stimuli. We note, however, that internal metabolic cues may still be involved in this case, as aversive odor conditioning requires food deprivation. Past studies show that insulin-like signaling mediates the response to starvation and that this is required for aversive odor learning (Cho et al., 2016, Lin et al., 2010). If so, this suggests the possibility of AWC mitochondria acting as an intersectional hub to integrate sensory information and internal metabolic states. Recent studies have shown that ablation of AWC or increase in mtROS in AWC induces mitochondrial unfolded response (UPR^MT^) in the intestine, further supporting the possibility that AWC activity may be attuned to metabolic cues (Dishart and Pender, 2024, Liu et al., 2024).

Mitochondrial-mediated secretion of peptides can be seen in other systems besides neurons. Calcium influx into mitochondria is a prerequisite for hormone secretion in endocrine cells, most notably insulin from pancreatic β cells (Quan et al, 2015). In β cells, glucose uptake and subsequent metabolic processes trigger mitochondrial calcium influx, as part of a process known as metabolism-secretion coupling (Maechler et al, 1997; Wiederkehr et al, 2011). Mitochondrial calcium influx produces “coupling factors” that together with other signals contribute to insulin release, among which include mtROS and H_2_O_2_ (Leloup et al., 2009, Pi et al., 2007). Similar to our study, treatment with antioxidants prevented insulin release, showing that hormone secretion and neuropeptide secretion share similar mechanisms.

### Regulation of Neuropeptide Release From Neurons

One of the outstanding questions in neuroscience is how multiple neurotransmitters are regulated within the same cell. In addition to the classical neurotransmitters and monoamines, neurons often co-express several neuropeptides, but whether they are co-released or controlled individually is not well understood. Single-cell sequencing technology is beginning to reveal the prevalence of the existence of multiple neuropeptides per single neurons (Anneser and Satou, 2024, Hanchate et al., 2020, Smith et al., 2019, Wolff et al., 2025). This raises the question of what kinds of mechanisms exist that would allow for release of different combinations of neuropeptides depending on specific situations and context.

If neuropeptides can be individually regulated, what kinds of mechanisms would allow for separate regulation of neuropeptide release from the same neuron? Our study suggests one possible mechanism of differential release. We show that the release of another highly expressed neuropeptide, NLP-3, does not respond to MCU activation. Similar selectivity in neuropeptide release was also observed in the AIY interneuron of *C. elegans*, where mtROS caused the release of the FMRFamide-like neuropeptide FLP-1 but not FLP-18 (Jia & Sieburth, 2021). What determines whether a neuropeptide is controlled by the mitochondria will be a topic with important implications.

### Neuronal Mitochondria and Behavior

Mitochondria carry out a range of functions, from energy production, calcium buffering, lipid biosynthesis, ROS signaling to signaling cell death. In cells with extreme structural polarity, such as neurons, this range of functions often serve many compartment-specific roles, adding to the complexity of their function. Understanding the varied roles of mitochondria in neurons will help illuminate its contribution to various psychiatric, neurological, and neurodegenerative diseases. One study that directly linked mitochondrial function to behavior showed that reduced function and altered structure of mitochondria in the nucleus accumbens (NAc) result in reduced social dominance in mice (Hollis et al, 2015). Conversely, infusion of NAD^+^ precursor to improve mitochondrial respiration abolished the social disadvantage (Hollis et al., 2015). Moreover, administering the anxiolytic diazepam also reverted the social disadvantage by boosting NAc mitochondrial function through the release of dopamine into the NAc (van der Kooij et al., 2018). Finally, this effect could be blocked by blocking complex I function in the NAc. With the advent of improved tools to monitor brain activity and mitochondrial function, similar mechanistic studies exploring the causative links between mitochondria and behavior will improve our understanding of the many facets that are required for normal brain function.

## CRediT authorship contribution statement

**Hee Kyung Lee:** Writing – review & editing, Writing – original draft, Investigation, Conceptualization. **Abe Gayle Santos:** Investigation. **Kyu-Sang Park:** Writing – review & editing, Writing – original draft, Supervision, Investigation, Funding acquisition, Conceptualization. **Kyoung-hye Yoon:** Writing – review & editing, Writing – original draft, Supervision, Investigation, Funding acquisition, Conceptualization.

## Declaration of Competing Interests

The authors declare that they have no known competing financial interests or personal relationships that could have appeared to influence the work reported in this paper.

## Appendix A Supplementary material

Supplementary material

Figure S1. AWC morphology in wild type and mcu-1 mutant worms. Figure S2. *EGL-4 nuclear localization in response to 60 minutes odor exposure occurs in both N2 and mcu-1 mutants*. Transgenic worms expressing GFP-tagged EGL-4 were exposed to 60 minutes butanone to measure the proportion of GFP in the nucleus (nuclear index, NI) (Cho et al., 2016). *P* values were determined by nonparametric Kolmogorov-Smirnov test (ns, not significant; **P* < .05; ***P* < .01). Figure S3. *Strong blue light is required for mito-miniSOG to cause mitochondrial loss and fragmentation.* GFP labeling of mitochondria in the AWC neuron show that exposure to ambient light is not sufficient to cause cell ablation (blue light-). Irradiation with intense blue light for 30 minutes resulted in severe loss and fragmentation of mitochondria when observed immediately after the treatment (blue light +). Scale bars indicate 20 μm.

## Footnotes

Appendix A Supplementary data associated with this article can be found in the online version at doi:10.1016/j.mocell.2026.100367.
