# Supplementary material for: Mitochondrial calcium modulates odor-mediated behavioral plasticity in Caenorhabditis elegans
Source: Mol Cells. 2026 May 4;49(7):100367. doi: 10.1016/j.mocell.2026.100367 (PMC13227190; doi:10.1016/j.mocell.2026.100367)
Supplement: Supplementary file 1 — Supplementary material [file mmc1.docx]

**Table S1.** All strains used for this study

| **Experimental Models: Organisms/strains** | **Source** | **Identifier** |
| --- | --- | --- |
| N2 | CGC | N2 |
| *mcu-1 (tm6026)* | NRBP |  |
| *mcu-1 (tm6026); okyEx101[rab-3p::mcu-1 + unc-122p::gfp]* | This study | KHY010 |
| *mcu-1 (tm6026); okyEx103[ceh-36p::mcu-1 + unc-122p:mCherry] (*indicated in study as strain #1) | This study | KHY129 |
| *mcu-1 (tm6026); okyIs100[hsp-16.2p::mcu-1 + unc-122p::mCherry]* | This study | KHY231 |
| *okyEx131[ceh-36p::mito-roGFP + unc-122p::mCherry]* | This study | KHY170 |
| *mcu-1 (tm6026); okyEx131[ceh-36p::mito-roGFP + unc-122p::mCherry]* | This study | KHY266 |
| *okyEx132[ceh-36p::nlp-1::Venus + unc-122p::mCherry]* | This study | KHY220 |
| *mcu-1 (tm6026); okyEx132[ceh-36p::nlp-1::Venus + unc-122p::mCherry]* | This study | KHY221 |
| *okyEx147[ceh-36p::tomm-20::miniSOG::SL2::mCherry]* | This study | KHY263 |
| *okyEx132[ceh-36p::nlp-1::Venus + unc-122p::mCherry], okyEx147[ceh-36p::tomm-20::miniSOG::SL2::mCherry]* | This study | KHY264 |
| *okyEx134[ceh-36p::nlp-3::Venus]* | This study | KHY265 |
| *nlp-1(ok1469)* | CGC (C. elegans knockout consortium) | RB1341 |
| *mcu-1(tm6026); nlp-1(ok1469)* | This study | KHY297 |
| *IskEx1553[ceh-36-Δ1p::GCaMP3 + unc-122p::dsRed]* | Gift from Dr. Kyuhyung Kim | KHK21690 |
| *okyEx163-ceh-36p::COX8(x4)::CaMPARI2(L398T) + unc-122p::mCherry]* | This study | KHY288 |
| *pyIs500[ofm-1p::GFP + odr-1p::DsRed + odr-3p::GFP::egl-4]* | Gift from Noelle L’Etoile | JZ500 |
| *mcu-1(tm6026); pyIs500* | This study | KHY239 |

**Table S2.** All primers used for this study.

| **Cloning Primers** | **Forward 5'-3'** | **Reverse 5'-3'** |
| --- | --- | --- |
| Prab-3 | tcgtcgCTGCAGATCTTCAGATGGGAGCAGTGGAC | tgatgaGGATCCTGCTTTTTTGTACAAACTTGTCATC |
| mcu-1 | ATGAGGAATGGCCGATGCT | TTACTTTTCAGCTTCCAAATTGGAT |
|  |  | attattGCTAGCTTACTTTTCAGCTTCCAAATTGGAT |
| Venus | aataGGTACCAGTAAAGGAGAAGAACTTTTCACTGG | tattGAATTCTTATTTGTATAGTTCATCCATGCCATGT |
| nlp-1 | aataGGATCCACATCAACTTGAGGCAACGATGA | aataGGTACCACGACGTCCCAATCCGACAAAG |
| nlp-3 | aataGGATCCATGAGCAAAATCGTCGCTTGC | aataGGTACCATAGTAATTTTCCAACATTTCATATCGATTG |
| miniSOG | aaatACCGGTGAGAAAAGTTTCGTGATAACTGATCCA | aaatGAATTCAAATGGTACCTTATCCATCCAGCTGCACTCC |
| tomm-20 mitochondria targeting sequence | attattGGATCCATGTCGGACACAATTCTTGGTTTC | aaatACCGGTTGCTCCAGCCTGGGCACGTC |
| SL2 | aatGGTACCCGCTGTCTCATCCTACTTTCACC | ataatCTCGAGAGCAGTTTCCCTGAATTAAAATTAGAAG |
| CaMPARI2 | aaatGCTAGCCATCATCCCACCACCATGGATC | aaatGAATTCTTACGTACGCAGGTCCTCCTCTG |
