# Supplementary material for: Mitochondrial calcium modulates odor-mediated behavioral plasticity in Caenorhabditis elegans
Source: Mol Cells. 2026 May 4;49(7):100367. doi: 10.1016/j.mocell.2026.100367 (PMC13227190; doi:10.1016/j.mocell.2026.100367)

Figure S1

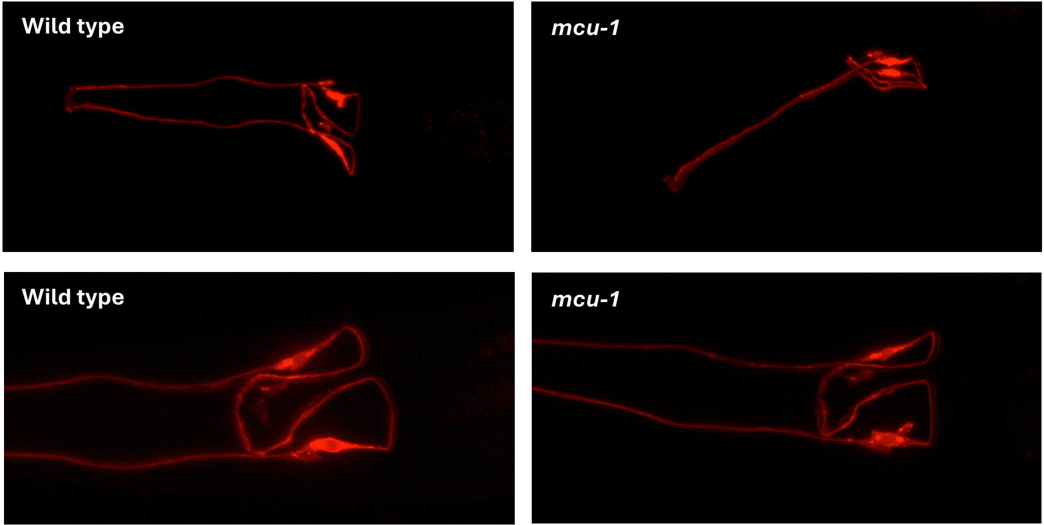

Figure S2

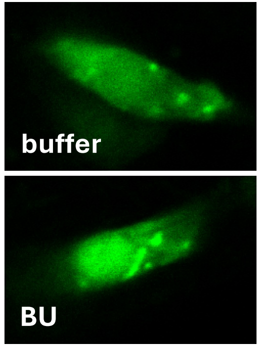

$$\text{Nuclear index (NI)} = \frac{F_{nucleus} - F_{cytoplasm}}{F_{nucleus} + F_{cytoplasm}}$$

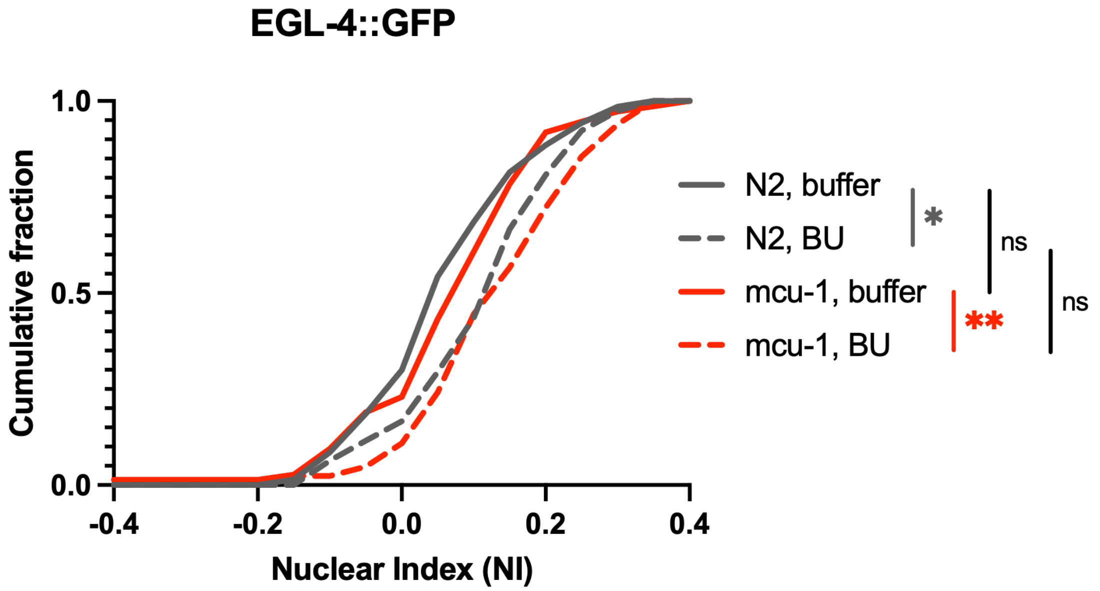

Figure S3

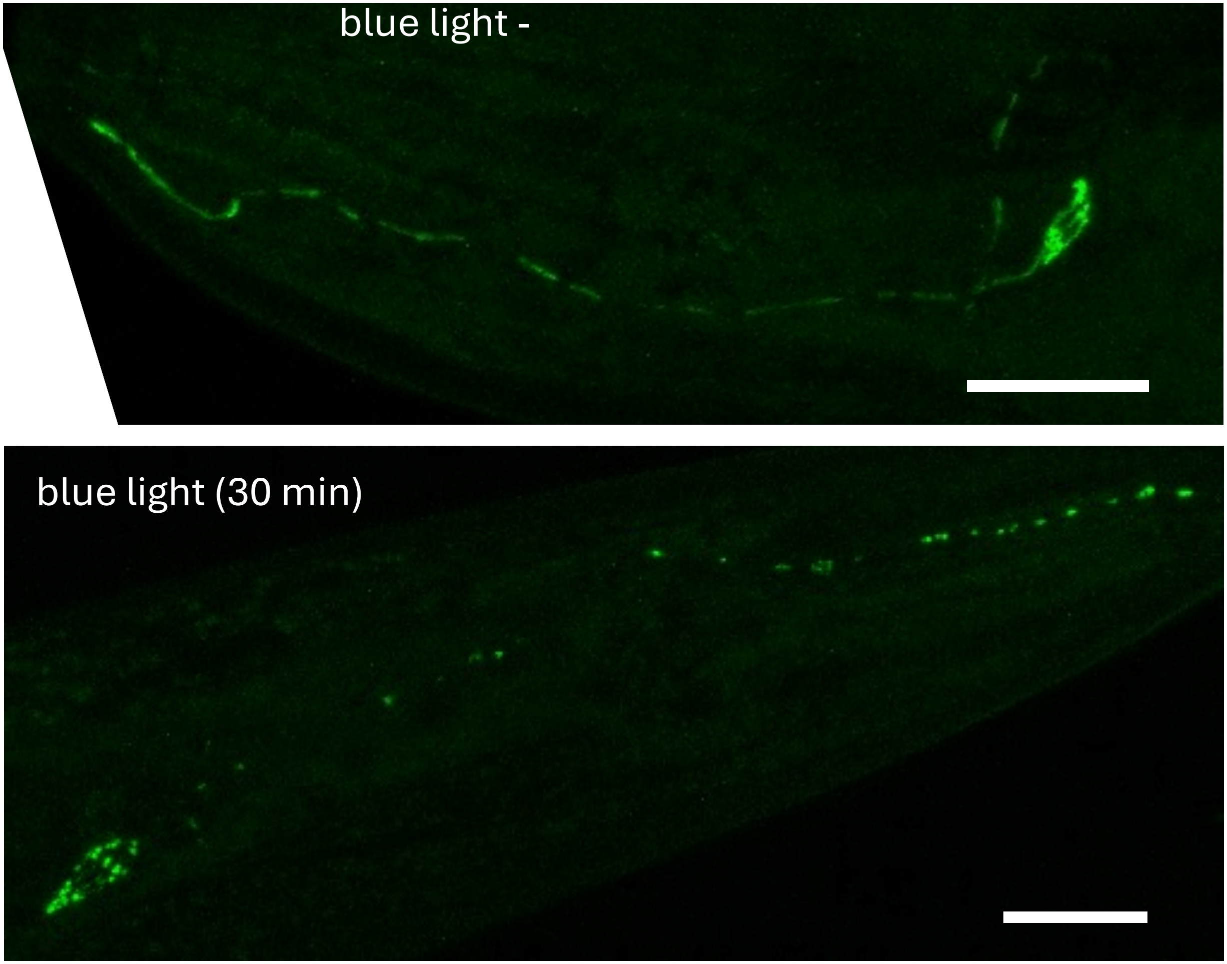

Supplement: Supplementary file 2 [file mmc2.pdf]
